# Probeless non-invasive near-infrared spectroscopic bioprocess monitoring using microspectrometer technology

**DOI:** 10.1007/s00216-019-02227-w

**Published:** 2019-12-04

**Authors:** Robert Zimmerleiter, Julian Kager, Ramin Nikzad-Langerodi, Vladimir Berezhinskiy, Frank Westad, Christoph Herwig, Markus Brandstetter

**Affiliations:** 1 RECENDT - Research Center for Non-Destructive Testing GmbH, Altenberger Straße 69, 4040 Linz, Austria; 2 Institute of Chemical, Environmental and Bioscience Engineering, TU Wien, Getreidemarkt 9/166, 1060 Vienna, Austria; 3 Camo Analytics, Gaustadalléen 21, 0349 Oslo, Norway; 4 Department of Engineering Cybernetics, Norwegian University of Science and Technology, O. S. Bragstads Plass 2D, 7034 Trondheim, Norway

**Keywords:** Near-infrared, *P. chrysogenum*, Non-invasive, In-line, Microspectrometer, Bioprocess monitoring

## Abstract

Real-time measurements and adjustments of critical process parameters are essential for the precise control of fermentation processes and thus for increasing both quality and yield of the desired product. However, the measurement of some crucial process parameters such as biomass, product, and product precursor concentrations usually requires time-consuming offline laboratory analysis. In this work, we demonstrate the in-line monitoring of biomass, penicillin (PEN), and phenoxyacetic acid (POX) in a *Penicilliumchrysogenum* fed-batch fermentation process using low-cost microspectrometer technology operating in the near-infrared (NIR). In particular, NIR reflection spectra were taken directly through the glass wall of the bioreactor, which eliminates the need for an expensive NIR immersion probe. Furthermore, the risk of contaminations in the reactor is significantly reduced, as no direct contact with the investigated medium is required. NIR spectra were acquired using two sensor modules covering the spectral ranges 1350–1650 nm and 1550–1950 nm. Based on offline reference analytics, partial least squares (PLS) regression models were established for biomass, PEN, and POX either using data from both sensors separately or jointly. The established PLS models were tested on an independent validation fed-batch experiment. Root mean squared errors of prediction (RMSEP) were 1.61 g/L, 1.66 g/L, and 0.67 g/L for biomass, PEN, and POX, respectively, which can be considered an acceptable accuracy comparable with previously published results using standard process spectrometers with immersion probes. Altogether, the presented results underpin the potential of low-cost microspectrometer technology in real-time bioprocess monitoring applications.

**Figure:**
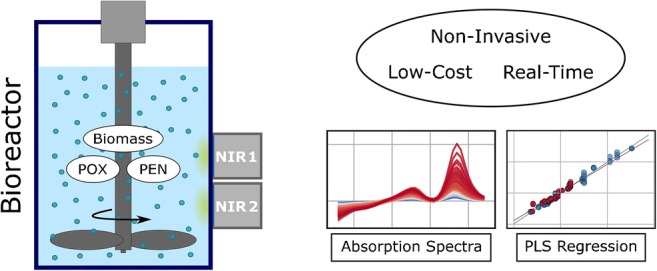
Graphical abstract

Graphical abstract

## Introduction

The precise control of bioprocesses is essential to achieve an optimum quality and yield of the desired product. A key to achieve this goal are analytical methods that provide real-time information of the current process state and critical process parameters. For some of these parameters such as pH, temperature, and dissolved oxygen concentration, real-time measurement techniques are readily available, while other parameters, such as substrate and product concentrations, usually require time-consuming offline measurements [1–3], which furthermore results in additional sources of analytical error due to sampling and sample preparation.

Many efforts have been made in the past to enable real-time monitoring of these parameters using different types of sensors including optical [4], capacitance [5], and ultrasound-based sensors [6] as well as nuclear magnetic resonance measurements [7]. Also, spectroscopic measurement techniques such as UV-Vis spectroscopy [8], fluorescence spectroscopy [9], and Raman spectroscopy [10] have already been utilized for bioprocess monitoring in the past.

One of the most promising sensing approaches for in-line application is near-infrared (NIR) and mid-infrared (MIR) spectroscopy, which, combined with multivariate data analysis, have been previously used for the real-time measurement of various process parameters [11, 12]. Spectral information is usually acquired using Fourier transform infrared (FTIR) spectrometers combined with a probe immersed into the bioreactor [13, 14]. The advantages of spectroscopic methods for bioprocess monitoring are manifold and include real-time capability, its non-destructive nature, easy maintenance, and the possibility for simultaneous determination of multiple target analytes in the complex fermentation broth. However, the commonly used FTIR spectrometers and measurement probes are costly and the probes usually have to be immersed into the fermentation broth, which makes sterilization of the probes a necessity.

With the recent advent of novel miniature spectrometer technology (“microspectrometer”) based on micro-electromechanical systems (MEMS), compact, robust, and cost-effective NIR spectrometers became available and significantly lowered the hardware costs for multiple NIR sensing applications [15]. This makes NIR spectroscopy much more attractive and creates a potential to enable completely new applications for the measurement technique. Different instrument technologies are available, with the most widespread being Fourier transform, Fabry-Pérot (FP), and dispersive spectrometers [16]. In recent years, the potential of microspectrometer technology has already been demonstrated in spectroscopy [17, 18], hyperspectral imaging [19], and compressive sensing [20] applications.

In this work, it will be demonstrated that FP-based microspectrometers can be utilized for real-time bioprocess monitoring, even without the need for an immersion probe via measuring through the glass wall of a bioreactor in reflection geometry. This enables a completely non-invasive measurement and therefore significantly increases the practicability and convenience of NIR process monitoring.

## Materials and methods

### *P. chrysogenum* fermentation and reference analytics

Fed-batch experiments using a spore suspension of an industrial *Penicillium chrysogenum* strain for penicillin production were performed in a 2.7 L parallel bioreactor system (Eppendorf AG, Germany). After increase in the pH level, which indicates the end of the batch process, 300 mL of cell broth was transferred to bioreactors filled with 1700 mL defined fed-batch media (for the detailed composition of batch and fed-batch media, the reader is referred to [21]).

Stirrer speed (350–850 rpm) and oxygen addition to pressurized air were used to keep the dissolved oxygen above 40%, while the aeration rate was kept constant at 1 vvm. During fermentation, the pH was sustained at 6.5 by addition of KOH and H_2_SO_4_ while the temperature was controlled at 25 °C. The supplied feeds were glucose (500 g/L), penicillin V precursor (80 g/L phenoxyacetate), and the nitrogen source (100 g/L (NH_4_)_2_SO_4_). The process was strictly glucose limited, whereas phenoxyacetate and nitrogen were kept at non-limiting concentrations by adjusting their feed rates.

Samples for offline reference measurements were taken every 8–10 h during the fermentation process. Determination of the penicillin V (PEN) and phenoxyacetate (POX) concentrations in the filtered broth was performed by high-performance liquid chromatography (HPLC) using a ZORBAX C-18 Agilent column and 28% acetonitrile, 6 mM H_3_PO_4_, and 5 mM KH_2_PO_4_ as an elution buffer. POX eluted after 2.75 min and was quantified between 0.00275 and 0.275 g/L. PEN eluted after 10.00 min and the calibration range was between 0.00468 and 0.468 g/L. For analysis, a 1:40 dilution of media samples with 1% citric acid was injected. For biomass determination, cells were separated from a 5 mL culture broth using centrifugation at 4800 rpm for 10 min at 4 °C, washed with 5 mL deionized water and dried at 105 °C. The remaining substance was then measured gravimetrically to get the amount of dried biomass.

### NIR spectroscopy

**Fig. 1:**
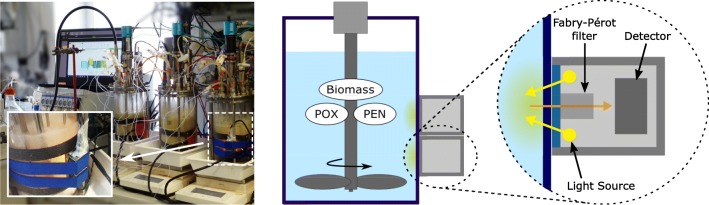
(Left) Photograph of the experimental setup, where the rightmost bioreactor is monitored using the MEMS-based microspectrometers (sensor 1 and sensor 2). The inset photograph shows a closer look on the two sensors attached to the glass. (Middle) Schematic drawing of the bioreactor with the attached sensors. (Right) Scheme of the key components of the microspectrometer

Robust, compact, and low-cost NIR Fabry-Pérot (FP) microspectrometers (NIRONE Sensors, Spectral Engines, Finland) were attached to the glass wall of the bioreactors before the fermentation process was started. Various types of FP microspectrometers covering different wavelength regions are available and the selection of the right wavelength range is crucial for extracting relevant information. NIR monitoring of biomass, PEN, and POX concentrations in the same fermentation process using a broadband Fourier transform NIR spectrometer coupled to an immersion probe was already conducted in the past [22]. Therefore, the published results were used to identify the most relevant wavelength regions for the analytes of interest. It was concluded that the most relevant available wavelength regions were 1350–1650 nm (“sensor 1”) and 1550–1950 nm (“sensor 2”). A photograph and a schematic drawing of the experimental setup are shown in Fig. 1. This setup enables a *probeless* and completely *non-invasive* acquisition of NIR spectra in reflection geometry. Spectra were taken every second and then averaged over approximately 60 s to lower the influence of short-lived disturbances such as air bubbles in the broth while still allowing for real-time monitoring of the fermentation process. As a light source, the built-in halogen lamps of the microspectrometers were used, which means no external light source was necessary to conduct the measurements. In between the two monitored fermentation processes, the spectral sensors were completely removed and reattached to the bioreactor, possibly resulting in slightly different sensor positions and/or orientations. The resulting differences in the recorded spectral signals are eliminated by the calculation of absorbance spectra using the spectrum before inoculation of the reactor with the *P. chrysogenum* batch culture for each fermentation process as reference as well as the applied spectral pre-processing.

### Multivariate data analysis and partial least squares regression

All spectra were pre-processed by calculating the 1st derivative and employing a 2nd-order polynomial fit on a window size of five using a Savitzky-Golay (SavGol) filter. For the monitoring of biomass concentration, additionally, a standard normal variate (SNV) normalization was applied. For each sensor, the entire spectral range was used for all calculations and modelling procedures. Principal component analysis (PCA) and partial least squares (PLS) regression were carried out in Unscrambler X 10.5.1 (Camo Analytics, Norway). All PLS models were fitted using data from one batch and validated using data from another, independent batch produced two months later. A total of 18 and 15 reference values for each analyte were available for the calibration and validation batch, respectively. The number of latent variables used in the PLS models was optimized using leave-one-out cross-validation.

## Results and discussion

**Fig. 2:**
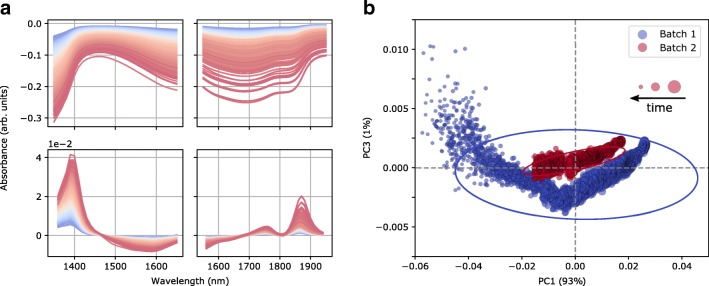
**a** Spectra used for multivariate calibration. (Top) Raw spectra. (Bottom) Pre-processed spectra (for details, see text). In the left column, spectra from sensor 1 and on the right spectra from sensor 2 are shown. The spectra are color coded and chronologically ordered from blue to red. **b** PCA of the pre-processed spectra of two independent batches. Scores on the third principal component (PC3) are plotted against scores on PC1. Dot size decreases with increasing time

Two full fermentation runs were pursued in order to test the suitability of MEMS-based microspectrometer technology for probeless monitoring of biomass, PEN, and POX in a *P. chrysogenum* batch fermentation. Figure 2a shows raw and smoothed (SavGol) 1st derivative spectra from sensor 1 and sensor 2 recorded over the course of the calibration batch. The most prominent changes in the pre-processed spectra were found around 1400 nm (sensor 1) and 1875 nm (sensor 2), which can be mainly attributed to CH_*x*_ and ROH oscillations in the second and first overtone regions, respectively [23]. This is in line with previous measurements done in a similar process environment [22]. On the other hand, the changes visible in the raw spectra are rather unspecific, and can be mainly attributed to scattering effects. This nicely underpins the importance of proper pre-processing in order to uncover the relevant information in the spectra. A principal component analysis (PCA) of the fused spectra of both sensors was undertaken in order to investigate if the data from the two batches are consistent (Fig. 2b). As expected, the time evolution of the batches is captured by the first principal component (PC1), which accounts for most of the observed variation (93%) in the data. Notably, the second batch trajectory starts on a slightly lower and ends on a higher PC1 score. This indicates a higher inoculum size and lower biomass in the beginning and the end of the batch, respectively, which is confirmed by the corresponding dry weight analysis (results not shown). On the other hand, the first batch displays high variability along PC3 towards the end of the batch, which can be attributed to elevated scattering effects towards higher optical densities (occurring at higher biomass concentrations).

**Fig. 3:**
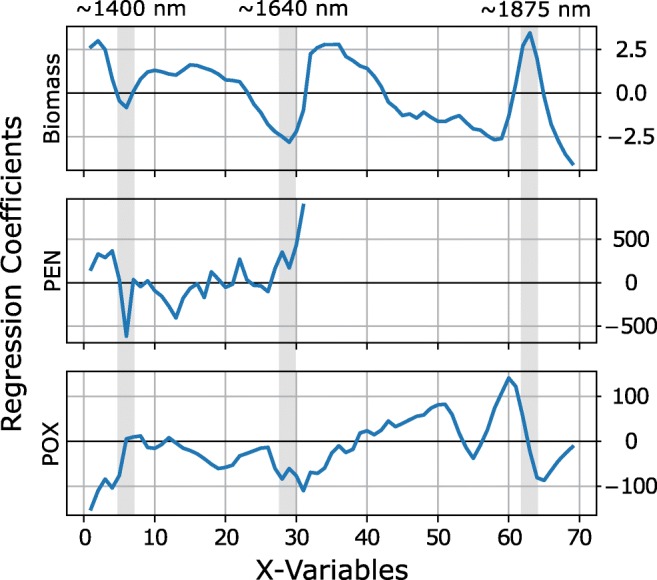
Regression coefficient vectors for the three determined analytes. *X*-variables represent the spectral axis with combined data from sensor 1 and sensor 2. Three exemplary wavelength regions showing the differences in the spectral responses are indicated in gray and the corresponding wavelengths are given

The pre-processed spectra from the first batch process, combined with the corresponding reference measurements, were used to establish PLS models for the prediction of biomass, PEN, and POX concentrations. Reference measurements were done in triplicates and three subsequently recorded spectra were used for each averaged reference value in order to facilitate the identification of potential outliers. However, in the data presented herein, no clear outliers were identified and all acquired data points were used for both calibration and validation. In order to compare the two sensors and identify the most suitable one, separate models were calculated for each sensor individually. In addition, models based on the fused spectra from both sensors were established. The regression coefficient vectors for biomass, PEN, and POX that yield the best predictive performance are shown in Fig. 3. The regression vectors for biomass and POX were calculated using spectral data from both sensors, utilizing three and four latent variables (LVs), respectively. For the calculation of PEN, the data from sensor 1 (i.e., the first 31 data points) was sufficient and the best results were achieved using four LVs.

The regression coefficient vector for biomass determination has clear extrema at the positions of strongest changes in the pre-processed spectra at around 1400 nm and 1875 nm (Fig. 2), which are indicated with a gray background in Fig. 3. The absorption at around 1400 nm has a negative and the absorption around 1875 nm has a strong positive contribution. Additionally, the extremum at 1640 nm is highlighted to emphasize the different spectral dependencies of the calculated regression vectors for PEN, POX, and biomass. This indicates that the models are responding to different chemical signatures in the spectral data, despite them showing similar concentration trends as shown below in Fig. 5. The differences in the spectral responses in the regression vectors fit nicely to the observed differences in the absorption spectra of PEN and POX in water published elsewhere [22]. The predictive performance for each of the established models as well as the number of latent variables (# LVs) is summarized in Table 1.

**Table 1:** PLS model statistics. The number of latent variables (# LVs), coefficient of determination (*R*^2^), root mean squared error of cross-validation (RMSECV), and root mean squared error of prediction (RMSEP) are indicated for single and fused sensor models. The best values for each analyte are highlighted in italics

Sensor	Stats	Biomass	PEN	POX
Sensor 1 (1350–1650 nm)	# LVs	2	*4*	2
	*R*^2^	0.96	*0.75*	0.89
	RMSECV (g/L)	2.19	*1.57*	0.47
	RMSEP (g/L)	10.51	*1.66*	0.95
Sensor 2 (1550–1950 nm)	# LVs	4	3	3
	*R*^2^	0.98	0.69	0.72
	RMSECV (g/L)	2.48	1.95	0.93
	RMSEP (g/L)	3.22	3.68	1.92
Sensor 1 + 2 (1350–1950 nm)	# LVs	*3*	2	*4*
	*R*^2^	*0.98*	0.43	*0.93*
	RMSECV (g/L)	*1.64*	1.38	*0.40*
	RMSEP (g/L)	*1.61*	2.95	*0.67*

**Fig. 4:**
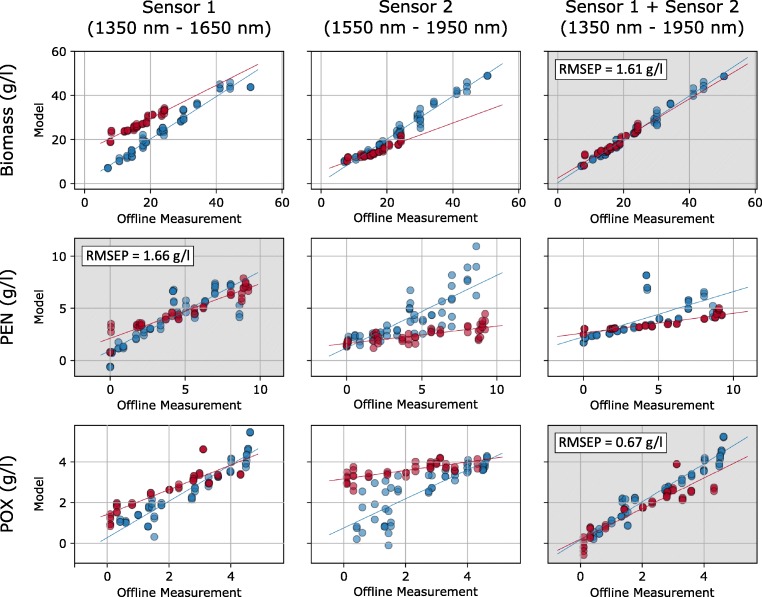
Model values vs. offline measured concentrations for the three investigated quantities (biomass, PEN, and POX). The data in the first, second, and third columns were obtained using spectral information from the first sensor, second sensor, and both sensors, respectively. Blue dots show the cross-validation of the calibration set and red dots show the predictions on the validation data set. The best PLS models are shown with a gray background with the achieved RMSEP values indicated in the graph

Except for PEN, the best correspondence between measured and predicted values in terms of coefficient of determination (*R*^2^) and cross-validation error (RMSECV) was obtained when fusing the spectra from both sensors achieving 0.98 and 0.93 (*R*^2^) and 1.64 g/L and 0.4 g/L (RMSECV) for biomass and POX, respectively. When tested on data from the second batch, the corresponding models achieved high accuracies for prediction of biomass and POX, clearly outperforming the models established using only the spectra from either one of the single sensors. In contrast, prediction of PEN was most accurate when using only the sensor operating in the 1350–1650 nm regime, whereas data fusion with the spectra from the second sensor yielded poor predictive performance irrespective of the number of LVs included in the model. This can also be seen in the graphs in Fig. 4 where the values given by the regression models are plotted against the corresponding reference measurements for both cross-validation (blue) of the calibration data and predictions for the validation batch (red).

A reasonably good RMSECV value of 2.19 g/L was also achieved for the PLS model with only spectral data from sensor 1 for the biomass content (upper left in Fig. 4), but when the model was applied to data from the validation batch, poor agreement between model and reference measurement was observed (RMSEP = 10.51 g/L). Upon a closer look on the data, it becomes clear that the main error in the model values stems from an offset and a slightly wrong slope. As has been shown elsewhere [24], additive PLS (aPLS) modelling can be used in a scenario like this to significantly reduce the errors of the model values by applying an additional PLS regression to the residuals of the initial regression model. Indeed, if aPLS is used to correct for the slight changes in measurement conditions between the first and second batch, greatly improved RMSEP values for the biomass content of about 1.56 g/L can be achieved (regression curves not shown). This value is on par with the RMSEP achieved with the PLS model using fused spectral data from both spectral sensors, but requires an additional modelling step.

**Fig. 5:**
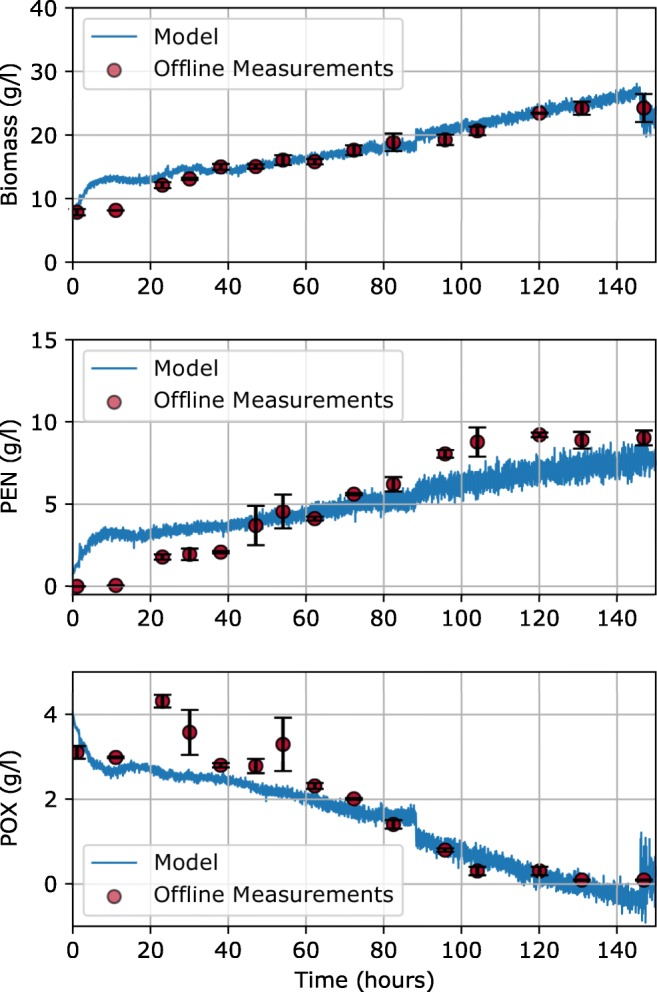
Values for biomass, PEN, and POX for the validation batch as a function of process time. The red circles represent the offline reference measurements and the blue line gives the values from the PLS model. The error bars for each offline measurement shown in black give the standard deviation of the three conducted measurements. For each of the analytes, the best performing model was utilized

Figure 5 shows the time-resolved predictions of the best performing models (highlighted in gray in Fig. 4) along with the offline reference values over the course of batch 2 (validation batch). It can be seen that biomass and PEN are overestimated especially in the first 20 h of the fermentation, while the penicillin V sidechain, POX, seems to be estimated correctly in this timeframe. The subsequent sudden increase in POX concentration, indicated by three of the measurements between 20 and 60 h, points towards possible outliers since they have quite a large estimated error and no significant POX addition occurred in this timeframe.

The normalized root mean squared error (NRMSE) of the models for the three analytes, biomass, PEN, and POX, was calculated by normalizing the RMSEP with the total range of the reference data. This calculation yielded values of 9.8%, 18.0%, and 15.9%, respectively.

In order to judge the performance of the established PLS models, the NRMSE of the validation batch was compared with the estimated error of the reference analytics. This error was assessed by analyzing the reference measurements from several similar batch processes and calculating the mean relative standard deviation for each value. This leads to relative errors of 5.4%, 7.3%, and 5.7% for biomass, PEN, and POX, respectively. The relative errors of the reference measurement follow a similar trend as the NRMSE of the established models. In both cases, PEN shows the largest while biomass shows the smallest relative error. By multiplying these values with the mean reference value for the validation batch, the absolute errors for this batch can be estimated to be around 0.86 g/L, 0.39 g/L, and 0.15 g/L for biomass, PEN, and POX, respectively.

It should be noted here, that since only one sample was taken from the bioreactor for each measurement, the sampling error, which is one of the main sources of error in analytical chemistry, is not considered in this estimation. Here, only the measurement error due to sample preparation and reference instrumentation is covered by the stated error bars. Therefore, this can only be seen as a lower boundary of the deviation from the offline measurement to the actual value of the analyte in the fermentation broth. The actual absolute errors of the presented method can thus be expected to be even lower.

When comparing the achieved RMSEP values to previously published prediction errors of models that were calculated using NIR spectra acquired with an invasive in-line measurement probe, the achieved values stack up even better. For example, an RMSEP value of 1.39 g/L for biomass in a fed-batch *Escherichia coli* process [14] and 2.62 g/L, 0.34 g/L, and 0.51 g/L for biomass, PEN, and POX, respectively, for a *P. chrysogenum* fermentation [22] were reported. Except for the prediction of the PEN concentration, the values achieved in this work are on par with the previously reported results from invasive NIR measurements.

## Conclusions and outlook

The potential of *non-invasive* NIR spectroscopic measurements in reflection geometry through the glass wall of a bioreactor for real-time bioprocess monitoring was successfully demonstrated. This was achieved by acquiring spectral data using novel NIR microspectrometer technology that is both low-cost and robust. Spectral data from two microspectrometer modules covering different wavelength ranges as well as offline reference data were used for calibrating PLS regression models for three different analytes (biomass, PEN, and POX) in a *P. chrysogenum* fed batch process. Validation of the established models was carried out using data from an independent batch process. Especially with regard to cost, size, and contamination risk, this approach is highly preferable over conventional NIR spectrometers connected to a measurement probe submerged into the fermentation broth while achieving similar performance. The reported approach is widely applicable and could give new insights into various different bioprocesses used in different industrial as well as scientific applications and allow for a cost-effective online monitoring and process control.

Comparison of different PLS models calibrated with single sensors and the fused spectral data of both NIR sensor modules showed that for two of the three analytes (biomass and POX), the model calculated with the fused spectral data showed the best performance. This hints towards the possibility to significantly improve the models when a third or fourth spectral sensor is used to widen the observed spectral range. This could also lead to better model performance for the determination of PEN concentration which was slightly worse than the one achieved previously via invasive measurements.

Another possibility would be to improve the performance of the multivariate analysis by using more elaborate regression methods than classical PLS. For example, domain invariant PLS (di-PLS) which can be useful to decrease influence on the model prediction quality stemming from changes in environmental conditions, instrumental response, or sample matrix [25], could be applied to the data. This however is subject of future research.

## Data Availability

All data generated and analyzed during the current study are available from the corresponding author on reasonable request.
